# The Cancer Prevention and Control Research Network

**Published:** 2004-12-15

**Authors:** Jeffrey R Harris, Pamela K Brown, Coughlin Steven, Katherine Wilson, Maria E Fernandez, James R Hebert, Jon Kerner, Marianne Prout, Randy Schwartz, Eduardo J Simoes, Carol White

**Affiliations:** Health Promotion Research Center, University of Washington Health Promotion Research Center; Mary Babb Randolph Cancer Center, West Virginia University, Morgantown, WV; Division of Cancer Prevention and Control, National Center for Chronic Disease Prevention and Health Promotion (NCCDPHP), Centers for Disease Control and Prevention (CDC), Atlanta, Ga; Division of Cancer Prevention and Control, National Center for Chronic Disease Prevention and Health Promotion (NCCDPHP), Centers for Disease Control and Prevention (CDC), Atlanta, Ga; Center for Health Promotion and Prevention Research, University of Texas Health Science Center at Houston School of Public Health, Houston, Tex; University of South Carolina, Columbia, the Hollings Cancer Center at the Medical University of South Carolina, and the Palmetto Health South Carolina Cancer Center, Columbia, SC; Division of Cancer Control and Population Sciences, National Cancer Institute, Bethesda, Mda; Department of Epidemiology, Boston University School of Public Health, Boston, Mass; New England Division, American Cancer Society, Boston, Mass; Division of Adult and Community Health, NCCDPHP, CDC, Atlanta, Ga; University of Kentucky Center for Prevention Research, Lexington, Ky

## Abstract

The Cancer Prevention and Control Research Network is a national network recently established to focus on developing new interventions and disseminating and translating proven interventions into practice to reduce cancer burden and disparities, especially among minority and medically underserved populations. Jointly funded by the Centers for Disease Control and Prevention and the National Cancer Institute, the Cancer Prevention and Control Research Network consists of sites administered through Prevention Research Centers funded by the Centers for Disease Control and Prevention. The five sites are located in Kentucky, Massachusetts, South Carolina, Texas, Washington State, and West Virginia. The Cancer Prevention and Control Research Network's intervention areas include primary prevention of cancer through healthy eating, physical activity, sun avoidance, tobacco control, and early detection of cancer through screening. The Cancer Prevention and Control Research Network uses the methods of community-based participatory research and seeks to build on the cancer-relevant systematic reviews of the *Guide to Community Preventive Services*. Initial foci for the Cancer Prevention and Control Research Network's research work groups include projects to increase screening for breast, cervical, and colorectal cancers; to promote informed decision making for prostate cancer screening; and to validate educational materials developed for low-literacy populations.

## Background

The Cancer Prevention and Control Research Network (CPCRN) is a federally funded, national network of academic, public health, and community partnerships that work together to reduce the burden of cancer, especially among those disproportionately affected. The CPCRN was initiated in October 2002, with funding from the Centers for Disease Control and Prevention (CDC) and the National Cancer Institute (NCI) as part of their effort to more effectively translate research into practice. The five CPCRN sites were selected through a competition among the CDC-funded Prevention Research Centers (PRCs). Three sites are operated by individual universities: the Universities of South Carolina, Texas-Houston, and Washington. Two sites are operated jointly by pairs of universities: Boston and Harvard Universities; and the University of Kentucky and West Virginia University. This paper introduces the CPCRN; outlines the context for its creation, along with its goals, structure, and operations; and summarizes progress to date.

## Context

Although the CPCRN sites carry out most of their work locally, the CPCRN is a national network and was developed in a national context. The CPCRN is a further step in efforts by two federal agencies, the CDC and the NCI, to translate research into practice with potential for reducing the cancer burden in the United States, especially among populations that are disproportionately affected. The context for the CPCRN, discussed in detail below, consists of four factors. First, the cancer burden in the United States remains high, and disparities in incidence and mortality persist. Second, one of the best opportunities to reduce these disparities is through community-based participatory research. Third, recently published syntheses of research, such as the CDC's *Guide to Community Preventive Services (Community Guide)* (1), suggest specific areas where carefully evaluated dissemination research is needed. Finally, the CDC's PRC Program (2), with its focus on community-based participatory research (CBPR) and translation, provides a unique combination of trained, experienced investigators and infrastructure to support a network like the CPCRN.

### Cancer burden in the United States

The creation of the CPCRN is, in part, a response to the growing magnitude of and persistent disparities in cancer burden. Cancer is the second leading cause of death in the United States as well as a leading cause of morbidity. Cancer accounts for one of every four deaths (3), and, in 2004, 563,700 people are expected to die of cancer. In 2004, 1,368,030 new cancer cases are expected to be diagnosed in the United States (3), not including carcinoma in situ or basal and squamous cell skin cancers. The top four cancer sites (with expected numbers of cases) are prostate (230,110), breast (217,440), lung (173,770), and colorectal (150,950). In addition, more than 1 million cases of basal and squamous cell skin cancer are expected to be diagnosed in 2004

Disparities in cancer incidence and mortality persist. For example, the incidence of cervical cancer is four times as high among Vietnamese women as it is among other Asian American and Pacific Islander women (3). Overall, cancer mortality among African American men is 1.4 times higher than among whites, and cancer mortality among African American women is 1.2 times higher than among whites (4). Additional disparities in cancer incidence and mortality rates across major racial and ethnic groups in the United States are highlighted in recent reports (3,5) and below in descriptions of the CPCRN sites.

Those disparities are the result of a complex array of economic, social, and cultural factors, and these factors are also reflected in disparities in preventive behaviors. For example, smoking prevalence is now highest among American Indian (38.6%) and Alaska Native (27.4%) men and women (3,5). Screening prevalence for colorectal cancer is the lowest among Hispanics and Latinos (3,5). To optimize the effect of cancer prevention efforts relative to expenditure, we need to be clear about 1) the efficiency of intervening on known risk factors early in the natural history of the carcinogenic process (e.g., reducing use of tobacco products, improving access to fresh fruits and vegetables), 2) the utility of various preventive services (e.g., screening), and 3) the willingness of communities to be engaged in cancer prevention and control.

### The importance of community-based participatory research

The CPCRN embraces the principles of CBPR (6) as core values. Research and evaluation developed with communities in a participatory way are more likely to reflect the needs, interests, and values of the community. Also, after research funding has ended, the results from such research and evaluation are more likely to be widely disseminated and the interventions to be sustained (7). The CPCRN has made a commitment in each of its sites to implement activities in a manner that is community-based and participatory to strengthen local resources and to build the capacity of community organizations to conduct and translate research. The CPCRN's commitment to CBPR is consistent with the strong and growing commitment of funding agencies to support this type of research partnership (8).

### The need for research dissemination and translation

The need for the CPCRN is highlighted by recent reports of both progress against cancer and remaining challenges in disseminating and translating knowledge gained from efficacy and effectiveness research. During the late 1990s, death rates from the four leading cancers — lung, colorectal, breast, and prostate — declined nationally and in most states (5). Two important prevention strategies have contributed to this decline but remain underused: 1) primary prevention by reducing risk behaviors and 2) early detection by increasing the use of screening services (3).

Both the NCI and the CDC have given high priority to bridging the dissemination gap. The NCI's Translating Research into Improved Outcomes program has identified the dearth of dissemination research as a key impediment to the adoption of evidence-based cancer control intervention. The program has also identified the need to expand research/practice partnerships as critical to both the adoption and evaluation of evidence-based interventions in public health and clinical practice settings (9).

The *Community Guide,* developed under the aegis of an independent, nonfederal Task Force on Community Preventive Services and maintained at the CDC, has provided a partial summary of the state of the art of community-based cancer prevention and control. Based on a systematic review of the literature, the *Community Guide* currently recommends 14 interventions aimed at increasing physical activity, reducing exposure to and use of tobacco, and reducing exposure to ultraviolet light (Table 1) (10-13). In addition to these recommendations, the Task Force has recently completed recommendations for increasing informed decision making regarding cancer screening (14) and plans to publish its reviews of interventions to increase cancer screening in 2005. 

The CDC has a ready dissemination outlet for proven intervention strategies through its state-based cancer prevention and control programs: the National Breast and Cervical Cancer Early Detection Program and the Comprehensive Cancer Control Program.

### The CDC Prevention Research Centers program

The prior investment of the CDC in its PRC program facilitated the creation of the CPCRN by providing a well-established administrative home for each of the five sites. In 1984, Congress created the PRC program at the CDC by authorizing the funding of academic health centers for innovative research and demonstration projects to prevent chronic disease. In 1986, the CDC established three PRCs for two years. Since then, the funding period has increased to five years, and the number of centers has grown to 28, which are located in 25 states.

Three aspects of the PRC program strengthen the ability of the participating CPCRN sites to design, implement, and evaluate cancer prevention research with immediate application to public health practice. First, the PRC peer-review mechanism involves researchers experienced with CBPR. The peer review process provides assurance that applications are scientifically sound and that the research conducted is of practical use to communities. Second, the program's focus on CBPR enhances the likelihood that the PRCs will evaluate acceptable and sustainable community-based interventions. Partnerships and collaborations of PRCs with various entities (e.g., businesses, community coalitions, grassroots organizations, private health care providers, state and local public health agencies, voluntary health organizations) increase the likelihood that PRCs will produce evaluations of interventions that are likely to be translated and sustained. Third, research conducted among the most disadvantaged and underserved populations in the nation provides the PRCs with the opportunity to evaluate the external validity of interventions among diverse populations, including the rural poor of Appalachia, African Americans in South Carolina, public housing residents in Boston, residents of the U.S.-Mexico border, and loggers and pulp-mill workers in Washington State.

## Methods

The overall goal for the CPCRN is to conduct community-based cancer prevention and control intervention and dissemination research that extends the knowledge base, addresses critical gaps, and leads to adoption, replication, implementation, and dissemination of successful programs in communities.

The CPCRN addresses gaps and builds on recommendations in the *Community Guide* by conducting site-specific and multisite intervention and dissemination research. The four specific research areas include 1) research on the effectiveness of community-based interventions for which evidence is insufficient to justify a *Community Guide* recommendation; 2) research replicating *Community Guide*-recommended interventions in populations and settings where they have not been adequately evaluated; 3) research on how to disseminate and implement *Community Guide*-recommended interventions into communities by public health and community-based organizations; and 4) evaluation of community programs to determine their effectiveness.

The initial funding of the CPCRN for a two-year period supported the development of both local network centers and the larger national network of the CPCRN sites. At both levels, expected outcomes are evidence of the networks' existence and viability, including mission and vision statements, short- and long-term objectives, and active working groups. Of special emphasis in the research arena are efforts to develop strong partnerships with communities bearing the greatest burden from cancer, where community-based participatory research projects are likely to contribute to the reduction and/or the elimination of disparities in cancer burden.

### Cancer Prevention and Control Research Network structure and operations

The CPCRN is a national network of five sites, each of which is a local network of academic, community, and other organizations with an interest in cancer prevention and research. The work of the CPCRN is led by a Coordinating Center that organizes, among other things, cross-site work groups on research topics of mutual interest to the sites, the CDC, and the NCI. The six sections that follow describe the work of the Coordinating Center and of each of the five local sites.

### Cancer Prevention and Control Research Network Coordinating Center

The University of Kentucky and West Virginia University share the coordinating center role for the CPCRN. The Coordinating Center guides discussions on developing research tools for community interventions in cancer prevention and control, organizes collaborative activities with CPCRN members and their partners, fosters relationships among CPCRN members and national/state/local partners to ensure that CPCRN objectives are being achieved, and provides leadership in developing and managing the CPCRN operational structure.

Specific activities include 1) developing and implementing a plan and system for effective communication among CPCRN centers; 2) implementing a collaborative planning process resulting in a seven-year plan for CPCRN research, dissemination, and evaluation; 3) implementing processes and procedures for encouraging PRCs to develop collaborative cancer prevention and control research projects; and 4) ensuring that external evaluation is conducted and is focused on the Coordinating Center's performance.

### Alliance for Reducing Cancer, Northwest

The site based at the University of Washington is the Alliance for Reducing Cancer, Northwest (ARC NW; available from: URL: http://www.arcnw.org). The mission of the ARC NW is to increase primary-preventive and early-detection behaviors to prevent and control cancer in the Puget Sound region, Washington State, and the Pacific Northwest. ARC NW is a collaborative effort among the University of Washington PRC, the American Cancer Society Great West Division; Fred Hutchinson Cancer Research Center; Group Health Cooperative of Puget Sound, a health maintenance organization; Public Health Seattle and King County, a local health department; the Puget Sound Neighborhood Health Centers, an organization of several community health centers in the region; Qualis Health, the Medicare quality improvement organization for Alaska, Idaho, and Washington State; the Washington State Department of Health; and the Weyerhaeuser Company, a large timber products company.

Data from the Washington State Behavioral Risk Factor Surveillance System reveal underuse of primary-preventive and early-detection behaviors. In 2002, 21% of Washingtonians aged 18 or older smoked, 15% were physically inactive during leisure time, 76% ate inadequate quantities of fruits and vegetables, and 60% were overweight or obese. Also in 2002, among appropriate age groups, 45% had never received a flexible sigmoidoscopy or colonoscopy, 40% had never received a fecal occult blood test, 26% had not received a mammogram within two years, and 13% had not received a Papanicolaou (Pap) test within three years (15).

One important factor in the underuse of these behaviors is the lack of support for prevention at the worksite and in employer-based health insurance. At the worksite, employers of all sizes reported in a 2001 national survey the following offerings: 11% offered fitness services and 5% offered tobacco-cessation services (personal communication, Maris Bondi, Partnership for Prevention, November 2003). Employers of all sizes nationwide reported in the same survey the following health insurance offerings: 80% covered mammograms, 79% covered Pap smears, 68% covered colorectal cancer screening, and 10% covered smoking cessation treatment that included both prescription medications and counseling.

The ARC NW focuses on employed populations and on underserved communities. Five current activities include 1) a pilot test, involving the Weyerhaeuser Company, of a policy intervention to promote primary prevention and early detection via the worksite and employment-based health insurance; 2) development of a work site-based, team-oriented intervention to promote primary prevention and early detection of cancer; 3) a pilot test of a tool to increase informed decision making regarding prostate cancer screening; 4) assistance to the Washington State Department of Health in designing and evaluating its colorectal and prostate cancer screening programs; and 5) a review of the literature regarding the quality of life after treatment of prostate cancer.

### Appalachian Cancer Research Consortium

The site based at the University of Kentucky and West Virginia University is the Appalachian Cancer Research Consortium (ACRC). The target population of the ACRC includes the poor, medically underserved, and primarily rural residents of West Virginia and the 51 counties in Appalachian Kentucky. The two universities have a long history of collaboration, with extensive experience in working with communities throughout Appalachia on critical health issues.

The U.S. Department of Health and Human Services considers the rural residents of Appalachia a "special population" (16). These residents tend to be older, poorer, less educated, and more likely to be uninsured than urban Americans. Rural communities have higher rates of chronic illness and disability and report poorer overall health status than urban communities (16). Residents of rural areas generally have fewer visits with physicians and lower levels of preventive care. In addition to factors related to rural health status and practices, there are systemic factors related to rural life that may contribute to less than optimal preventive care (17). These factors include lack of public transportation, lack of health care providers, and lower levels of community services.

As a result, West Virginia and the Appalachian regions of Kentucky have higher total cancer mortality rates than the national average (18,19). Both states rank among the top 10 U.S. states for total, male, and female cancer mortality. Lung cancer is a significant problem for residents, accounting for approximately 30% of all cancer deaths in West Virginia and Kentucky and resulting in a higher lung cancer mortality rate than the U.S. rate. Kentucky and West Virginia have invasive cervical cancer incidence and mortality rates that are significantly higher than the U.S. rates. West Virginia and Appalachian Kentucky also have higher colorectal cancer mortality rates than the United States and Appalachia as a whole. Breast cancer mortality rates are similar to national rates, but breast cancer mortality in several rural counties exceeds the national rate by more than 50%.

The ACRC focuses its efforts primarily on four cancer sites — lung, cervix, colorectal, and breast — with high disease burden, high behavioral risks, and high importance to community members in the region. Current activities of the ACRC include 1) developing a standardized assessment tool to evaluate readability, format, illustrations, and content of cancer prevention and control materials; 2) developing a protocol for colorectal cancer intervention for men and women aged 50 and older; and 3) conducting work site focus groups to identify barriers to colorectal screening for public employees aged 50 and older.

### Latinos in a Network for Cancer Control

The site based at the University of Texas (UT) is Latinos in a Network for Cancer Control (LINCC; available from: URL: http://www.sph.uth.tmc.edu/research/lincc). The mission of the LINCC is to reduce cancer-related health disparities among Hispanics/Latinos through community-based intervention, replication, and dissemination research. LINCC is a collaboration among 1) academic researchers at the UT School of Public Health, the UT M.D. Anderson Cancer Center, and the Baylor College of Medicine; 2) cancer control organizations, including the American Cancer Society, Cancer Information Service, Sanchez Cancer Center, Texas Cancer Council, Texas Comprehensive Cancer Coalition, and the Texas Department of Health; and 3) community-based organizations, including the Center for Border Health Research, Hispanic Health Coalition, Migrant Health Promotion, the National Center for Farmworker Health, and the Racial and Ethnic Approaches to Community Health coalition.

Hispanics/Latinos in Texas account for approximately 25% of the total U.S. Hispanic population and 32% of the total Texas population (20). Along the U.S.-Mexico border where LINCC has focused its initial research efforts, Hispanics comprise roughly 80% of the population (21). Many border residents experience high rates of poverty and live in *colonias*, unincorporated areas where environmental pollution, inadequate wastewater systems, and inadequate access to public drinking water compound socioeconomic influences on health behavior.

Hispanics in the United States experience higher incidence rates of cervical cancer per 100,000 (16.3) compared with non-Hispanics (7.8) and higher rates of mortality per 100,000 (3.7 compared with 2.6) (22). Along the U.S.-Mexico border, the disparity is even greater: the incidence rate of cervical cancer per 100,000 among Hispanics (18.7) is higher than the rate among non-Hispanics (8.2), and the mortality rate among Hispanics (6.2) is higher than the rate among non-Hispanics (3.4) (23). In addition, Hispanics have lower rates of cancer screening. Only 27% of the older Hispanic adults in Texas reported having a recent fecal occult blood test for colorectal cancer (compared with 34% among non-Hispanic whites), and only 50% reported regular mammography use (compared with 60% for non-Hispanic whites) (24). Use of Pap tests for cervical cancer screening among Hispanics (83%) was also lower compared with non-Hispanic whites (87%) (22).

Current LINCC activities include 1) new research on factors influencing colorectal cancer screening among Hispanics and the development of a community-based intervention to increase this screening; 2) research on informed decision making for prostate and colorectal cancer screening; 3) an evidence review and new research on lay health-worker- (*promotora-*) based interventions for increasing cancer screening; and 4) research on the effectiveness of small media interventions to increase cancer screening. Another major focus of LINNC is to identify important factors and effective strategies for replicating and disseminating effective cancer control interventions in Hispanic communities. To this end, LINCC is conducting research on the replication and dissemination of an evidence-based, effective breast and cervical cancer screening intervention for Hispanic women: *Cultivando la Salud* (Cultivating Health) (25).

### Massachusetts Cancer Prevention Community Research Network

The site based at Boston and Harvard Universities is the Massachusetts Cancer Prevention Community Research Network (MCPCRN). The MCPCRN's mission is to foster a network of partnerships among cancer prevention researchers and community collaborators to support CBPR and to reduce social disparities in cancer risk. The MCPCRN is a collaboration of the Dana-Farber/Harvard Cancer Center Risk Reduction Program, the Harvard Prevention Research Center (HPRC), and the Boston University Prevention Research Center, with participation from the American Cancer Society's New England Division and the Massachusetts Cancer Control Coalition.

Massachusetts has 6.5 million residents, 82% of whom are non-Hispanic white (26). In Boston, however, because of recent immigration, non-Hispanic whites are no longer in the majority (27). The total cancer incidence rate per 100,000 in Massachusetts (501.2) is higher than the national rate (468.9) in the Surveillance, Epidemiology, and End Results Program, and so are the incidence rates for prostate, breast, lung, and colorectal cancers (4,28). The total cancer mortality rate per 100,000 in Massachusetts (211.3) is just slightly higher than the U.S. rate (206.0) (4,28); and colorectal and breast cancers are the major contributors with higher mortality rates. Smoking rates have fallen to less than 19%; 20.8% of the population is sedentary, and 54.4% is overweight or obese (15).

To reduce these risks, the MCPCRN is approaching four priority community sectors: 1) schools and youth, 2) work sites and labor unions, 3) health care providers, 4) and low-income housing. Among schools and youth, the HPRC faculty direct a range of school and community-based research to improve youth nutrition and physical activity. Work sites and union partners include the Massachusetts AFL-CIO (American Federation of Labor-Congress of Industrial Organizations), the Massachusetts Coalition on Occupational Safety and Health, and individual local unions. Approximately 50 community health centers, many with strong ties to MCPCRN partners, facilitate access to health care providers in Massachusetts. The cost of housing in Massachusetts ranks third nationally; MCPCRN collaborators have identified more than 100 housing developments in Boston, Cambridge, and Somerville as potential partners.

The MCPCRN's current objectives are to strengthen ties with communities and to conduct pilot and developmental studies as a foundation for future research. An upcoming conference on CBPR will emphasize engaging community organizations in cancer prevention research opportunities. Collaborative community efforts support Health Ambassadors for African American and African immigrant women in Boston and train tobacco advocates in housing developments. Developmental research includes a work site protocol to increase informed decision making for prostate cancer screening; materials to promote timely follow-up for abnormal mammograms among low-income, ethnic minority women; and methods to improve decision making on colorectal cancer screening. Pilot studies include an intervention aimed at weight reduction and increased physical activity through the Young Men's Christian Association and data collection in low-income housing developments.

### South Carolina Cancer Prevention and Control Research Network

The site based at the University of South Carolina is the South Carolina Cancer Prevention and Control Research Network (SCCRN). The SCCRN was created to address the large and growing cancer burden among African Americans living in South Carolina. Its aim is to serve the entire state, with a population of just more than 4 million people, comprising an area of 31,000 square miles, and ranging from a long, broad coastal plain to the Piedmont region of southern Appalachia. The SCCRN builds on a strong network of existing programs that have coalesced recently in the South Carolina Cancer Alliance (SCCA), which consists of more than 750 institutional and individual members. The constituent bodies of the SCCA include the South Carolina Department of Health and Environmental Control and numerous grassroots organizations in addition to all academic, clinical care, and nongovernmental organizations with cancer-related missions.

South Carolina is a relatively rural state, with very high (>40%) African American representation in rural areas. It is also a poor state, where the average personal income is about 81% of the national average (29). Cancer rates of African Americans, who represent 31% of South Carolina's total population, diverge from the U.S. average, in many instances markedly (4,30). For example, prostate cancer incidence among African American men in South Carolina is more than 70% higher than in white men, whereas the difference is 55% nationally (30). Nearly all cancers have higher mortality in South Carolina than in the United States as a whole (4). Illustrative of the pattern of increased mortality, breast cancer incidence in South Carolinian African American women is 18% lower than the incidence in white women (as opposed to being 15% lower nationally), but mortality is 47% higher (vs the national differential of 32%) (4,30).

Research at the SCCRN focuses on investigating ways to implement programs that complement existing cancer prevention and control infrastructure and through which we can anticipate risk reduction based on changes in individual and organizational behavior. The SCCRN focuses on breast, cervix, colorectal, oropharyngeal, prostate, and thoracic cancers. Ongoing projects include 1) investigation of small media approaches to increase breast and cervical cancer screening in low-income, rural women at highest risk of aggressive forms of these cancers, 2) research on informed decision making for prostate cancer screening and treatment, and 3) identification of geographical determinants of prostate cancer. Formative work includes 1) exploration of a community-based, statewide program of research in oral cancer precancerous lesions, 2) development of a mammography registry to understand patterns of use in low-income, predominantly African American populations, and 3) a church-based participatory intervention of lay health advocate-delivered cancer education and referral.

## Consequences

In its first year of operation, the CPCRN has focused on a strategic planning process. From the beginning, community partners from each of the five sites have played strong and active roles in these processes. The strategic planning process produced vision and mission statements; a set of operating structures, principles, and plans; and four research work groups (see below).

### Vision statement

Communities and researchers working together to reduce the burden of cancer, especially among those disproportionately affected.

### Mission statement

The mission of the CPCRN is to conduct cancer prevention and control research that extends the knowledge base, addresses critical gaps, and leads to adoption, replication, implementation, diffusion, and evaluation of successful programs in communities.

### Operating structure, principles, and plans

The CPCRN has developed a governing structure with a steering committee, guiding principles, and a seven-year strategic plan. Further information on each of these documents is available from: URL: http://ukprc.uky.edu/CPCRN/home.htm.

### Research work groups

The strategic planning process also suggested the development of work groups to initiate and carry out CPCRN research. The CPCRN currently has work groups focusing on 1) screening for breast and cervical cancers; 2) screening for colorectal cancer; 3) informed decision making and quality-of-life issues for prostate cancer screening and treatment; and 4) validating low-literacy educational and media materials. The work groups involve cross-site collaboration among scientists from the network centers and have established research goals (Table 2).

## Interpretation

The CPCRN represents a new and innovative approach for addressing the challenge of identifying effective interventions and promoting dissemination and adoption of these interventions into communities. The CPCRN sites are geographically distributed across the nation, enhancing opportunities to develop community partnerships and to conduct community-based assessments, evaluation, and research with populations that represent nearly all types of medically underserved racial and ethnic groups in the continental United States. A strong commitment to CBPR increases the likelihood that CPCRN research will benefit the underserved communities in greatest need. The CPCRN also provides an opportunity for the sites to collaborate in addressing research gaps, including dissemination research and research translation, and to build on recommendations provided in the *Community Guide.* Finally, the location of the CPCRN within the CDC's PRC program enables its research findings to be easily translated, both nationally and locally, through long-existing partnerships with other prevention organizations.

Fostering the optimal results from the CPCRN will require that its members maintain a delicate balance between coordinated, centralized efforts and retaining and enhancing the critical, locally responsive nature of its individual members. Within the tension between centralization and decentralization lies the exciting opportunity to create new strategies for successfully reducing the burden of cancer, especially among those disproportionately affected.

## Figures and Tables

**Table 1:** Cancer Prevention Interventions Recommended by *the Guide to Community Preventive Services *(10-12)

**Increasing Physical Activity**	**Reducing Tobacco Use and Exposure to Environmental Smoke**	**Reducing Exposure to Ultraviolet Light**
Behavioral and social approaches Individually adapted programs School-based physical education Group programs that foster social support Environmental and policy approaches Enhanced access to facilities, with informational outreach Stair-use reminders Informational approaches Community-wide campaigns Stair-use reminders	Increasing cessation Increasing the price of tobacco products Mass media campaigns Provider reminders Reducing treatment out-of-pocket costs Telephone quit lines Reducing exposure to environmental tobacco smoke Smoking bans and restrictions Reducing initiation Increasing the price of tobacco products Mass media campaigns	Setting-specific approaches Primary schools: education and policies Recreation/tourism: education and policies

**Table 2:** The Cancer Prevention and Control Research Network (CPCRN) Research Work Groups and Their Goals^a^

**Work Group**	**Research Goals**
**Colorectal cancer**	Develop a protocol for a community-based intervention trial to increase colorectal cancer screening and promote informed decision making for colorectal cancer screening among Hispanics. Develop a protocol for implementing an intervention to increase colorectal cancer screening among men and women aged 50 and older.
**Breast and cervical cancers**	Design a small-media community-intervention trial to increase the use and awareness of the CDC’s National Breast and Cervical Cancer Early Detection Program among program-eligible African American women. Replicate small-media interventions to increase breast and cervical cancer screening among Hispanics (both farm-working and non-farm-working populations) living in the Texas-Mexico border area.
**Prostate cancer**	Develop and pretest a work site intervention protocol to increase informed decision making for prostate cancer screening among men aged 50 and older. Develop an interactive and innovative decision-making tool to promote informed decision making for prostate cancer screening among men aged 40 to 70. Conduct and write a review of available literature regarding the effect of treatment on health-related quality of life among prostate cancer survivors, with an emphasis on African American men. Identify a care provider network that serves African American men and supports informed and shared decision making for prostate cancer screening; assess the network’s acceptance and perception of the usefulness and relevance of NCI materials for informed decision making, and field test these materials with African American men.
**Low-literacy materials validation**	Provide CPCRN sites and others with an extensive collection of tested materials for individuals who are among several minority and ethnic populations and have limited reading skills, and develop tools the CPCRN sites can use to validate materials. Conduct a review of existing materials that address the need for timely follow-up of mammographic abnormalities among low-income ethnic minority women and, where needed, adapt materials to better meet the needs of these women.

a CDC indicates the Centers for Disease Control and Prevention; NCI indicates National Cancer Institute.
